# Neural Control of Cardiovascular Function During Exercise in Hypertension

**DOI:** 10.3389/fphys.2018.01829

**Published:** 2018-12-20

**Authors:** Maryetta Dombrowski, Joseph Mannozzi, Donal S. O’Leary

**Affiliations:** Department of Physiology and Cardiovascular Research Institute, Wayne State University School of Medicine, Detroit, MI, United States

**Keywords:** hypertension, central command, arterial baroreflex, exercise pressor response, dynamic exercise

## Abstract

During both static and dynamic exercise hypertensive subjects can experience robust increases in arterial pressure to such an extent that heavy exercise is often not recommended in these patients due to the dangerously high levels of blood pressure sometimes observed. Currently, the mechanisms mediating this cardiovascular dysfunction during exercise in hypertension are not fully understood. The major reflexes thought to mediate the cardiovascular responses to exercise in normotensive healthy subjects are central command, arterial baroreflex and responses to stimulation of skeletal muscle mechano-sensitive and metabo-sensitive afferents. This review will summarize our current understanding of the roles of these reflexes and their interactions in mediating the altered cardiovascular responses to exercise observed in hypertension. We conclude that much work is needed to fully understand the mechanisms mediating excessive pressor response to exercise often seen in hypertensive patients.

## Introduction

**FIGURE 1:**
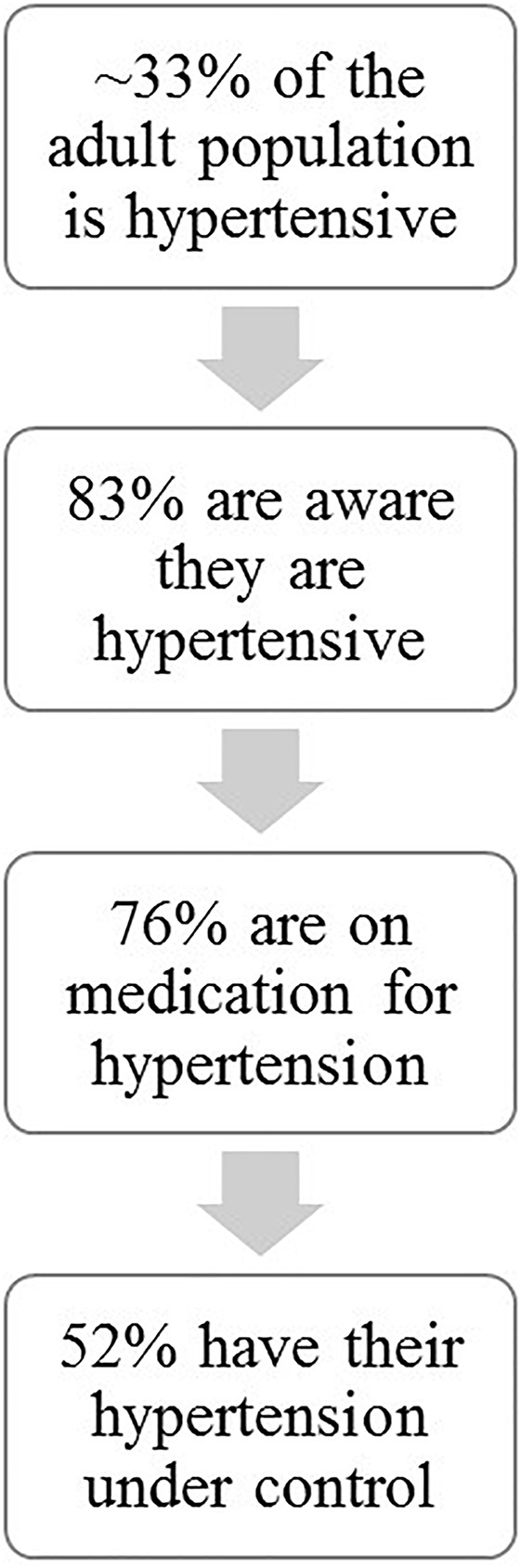
Incidence of hypertension.

Hypertension is one of the major pathological diseases of our time affecting approximately 1/3 (29.1%) of the adult population. Only 83% of these individuals are aware that they are hypertensive and of these, only 76% are medicated and despite multiple available therapeutic interventions only about one-half of treated individuals have their hypertension under control (Nwankwo et al., 2013) (Figure 1). Current extrapolations predict that by 2030 an additional 27 million people will be hypertensive (Kirkland et al., 2018). HTN has a myriad of risk factors: obesity, sedentary lifestyle, high salt diet, high alcohol intake, insulin resistance, low potassium intake, low calcium intake, stress, sex, and age all can contribute in concert or alone in the disease development and pathology (Carretero and Oparil, 2000). Excessive activation of the sympathetic nervous system may play a role in hypertension (Head, 1995). Although, regular moderate exercise can reduce the subsequent resting sympathetic activity in hypertensive individuals, several studies have shown that during exercise sympathetic activity may increase excessively thereby increasing risk factors for adverse events including myocardial ischemia, arrhythmia, myocardial infarctions, and sudden cardiac death (Hoberg et al., 1990; Mittleman et al., 1993). Indeed The American College of Cardiology/American Heart Association 2002 guidelines for high blood pressure warns that individuals with uncontrolled hypertension (>200 mmHg systolic and/or >110 mmHg diastolic at rest) should not undergo exercise stress tests due to possible dangerously large increases in arterial blood pressure (Gibbons et al., 2002). Similarly, the Journal of Nuclear Cardiology and The American Family Physician guidelines considers systolic blood pressure above 230 mm Hg and diastolic above 115 mm Hg during exercise as contraindications to continue these tests (Darrow, 1999; Henzlova et al., 2016). Clearly, pathological increases in arterial pressure during these tests present potential risks for adverse events such as myocardial ischemia, arrhythmia, myocardial infarction, and stroke. The mechanisms mediating these exaggerated increases in sympathetic activity in hypertensive patients are not well-understood and are clinically important. This review summarizes current understanding of the roles of three major mechanisms thought to control autonomic outflow during exercise in hypertension: central command, arterial baroreflex, and skeletal muscle afferents.

## Central Command

The initiation of exercise elicits immediate changes in autonomic outflow which have been in part ascribed to feed-forward reflex effects of the volition to exercise, termed central command. This reflex likely contributes importantly to the immediate partial reductions in parasympathetic activity to the heart causing a rapid tachycardia. Sympathetic activity can also increase with activation of central command. To our knowledge there have been no studies investigating whether neural control of cardiovascular function during exercise by central command is altered in patients with hypertension. However, Liang et al. (2016) showed in decerebrated rats that electrical stimulation of the mesencephalic locomotor region in spontaneous hypertensive rats (SHRs) had greater pressor and heart rate (HR) responses when compared to normotensive rats indicating that “central command” maybe exaggerated in hypertension.

## Skeletal Muscle Afferents

Skeletal muscle contains afferents that are both mechanosensitive (predominantly group III afferents) and sensitive to the metabolic environment (predominantly group IV afferents) although some afferents are polymodal (Kaufman et al., 1983, 1984; Kaufman and Rybicki, 1987). Activation of these afferents can elicit a powerful pressor response. Previous studies have concluded that the pressor, HR, and renal sympathetic nerve activity (RSNA) responses to stimulation of both mechanosensitive and metabosenstive skeletal muscle afferents are exaggerated in SHR compared to the normotensive controls (Leal et al., 2008; Mizuno et al., 2011a; Liang et al., 2016). In addition, a recent study (Barbosa et al., 2016) showed in humans that the exaggerated pressor response to leg exercise could be normalized by blockade of leg afferents via intrathecal fentanyl. Although the drug also lowered resting arterial pressure, these results strongly suggest that the exaggerated pressor response to exercise in hypertension stems, in part, from activation of skeletal muscle afferents.

### Muscle Mechanoreflex

In SHR, blockade of mechanoreceptors reduced the increases in mean arterial pressure (MAP), RSNA and HR when compared to control responses (Mizuno et al., 2011b) indicating that the muscle mechanoreflex is playing an important role in the exaggerated cardiovascular responses to exercise in hypertension. These studies (Mizuno et al., 2011a,b) provide some support for the role of the mechanoreflex, however, all of these studies were done either under anesthesia or in a decerebrated animal models. In a study using conscious humans Choi et al. (2013) observed that the pressor response to static forearm contraction was exaggerated in pre-hypertensive subjects whereas the pressor response to only mechanoreflex activation (via stretching of lower leg muscles) was only seen to be greater if expressed as absolute increases in pressure, % changes were not different.

### Muscle Metaboreflex

Multiple studies have been conducted in order to elucidate whether hypertension affects the strength and mechanisms of the muscle metaboreflex although the conclusions have been varied. Delaney et al. (2010) showed that hypertensive individuals had accentuated increases in mean arterial blood pressure and muscle sympathetic nerve activity (MSNA) in response to hand grip exercise when compared to normotensive individuals, whereas the responses to the cold pressor test were not different indicating that the accentuated metaboreflex responses likely were not due to generalized increases in sympatho-excitatory reflexes. Furthermore, these exaggerated responses were maintained during post-exercise circulatory occlusion (PECO) – a setting that isolates any metaboreceptor activation during the recovery from exercise. These results indicate that the accentuated responses to hand grip exercise may be due to accentuated muscle metaboreflex activation. Furthermore, Chant et al. (2018) found that the enhanced pressor response to exercise and metaboreflex activation still occurred in medicated hypertensive patients whose pressure was deemed controlled at rest. Studies in decerebrated rats have shown that chemical activation of skeletal muscle afferents with capsaicin leads to exaggerated RSNA (Mizuno et al., 2011a) and pressor responses (Leal et al., 2008; Mizuno et al., 2011a) in SHR when compared to Wistar-Kyoto (WKY) normotensive controls. Blockade of purinergic receptors in hypertensive individuals during PECO reduced MSNA burst frequency more when compared to normotensive individuals, suggesting that the purinergic receptors are playing a role in the sympathoexcitation occurring during muscle metaboreflex activation (Greaney et al., 2015b). In addition, women with a positive family history of hypertension had greater pressor and MSNA to several stressors such as the cold pressor test, isometric hand grip, and PECO when compared to women with no family history of hypertension (Greaney et al., 2015a) suggesting there may be a genetic component contributing to these accentuated responses.

In contrast there are several studies that have shown that muscle metaboreflex induced cardiovascular responses are reduced in hypertension. Rondon et al. (2006) found that the increases in MSNA in response to moderate handgrip exercise were not sustained during PECO whereas in normal individuals this sympatho-activation is sustained in PECO. A study by Ranadive et al. (2017) showed that there was no differences in muscle metaboreflex responses between women with a history of hypertensive pregnancies when compared to women that had normotensive pregnancies. Studies in conscious dogs concluded that metaboreflex-induced increases in MAP, cardiac output (CO), stroke volume (SV) and HR were reduced in dogs after induction of hypertension (Sala-Mercado et al., 2013) and that these attenuated responses were less sustained during PECO (Spranger et al., 2015). Previous studies in canines and humans have shown that when cardiac function is impaired the mechanisms mediating the metaboreflex pressor response during submaximal dynamic exercise “switch” from primarily increases in CO to primarily peripheral vasoconstriction (Hammond et al., 2000; Kim et al., 2005a; Crisafulli et al., 2011; Sala-Mercado et al., 2013). Furthermore, a recent study in canines demonstrated that metaboreflex activation during dynamic exercise elicits beta receptor-mediated peripheral vasodilation likely via epinephrine release from the adrenal gland (Kaur et al., 2015b). However, in heart failure (HF) this response appears to be abolished (Kaur et al., 2015b). In the study by Spranger et al. (2015) the authors noted a hint of this shift in the mechanisms mediating the metaboreflex in the animals after induction of hypertension inasmuch as the small rise in peripheral vascular conductance during metaboreflex activation often noted in normal animals was abolished after induction of hypertension. This could indicate greater peripheral sympatho-activation during metaboreflex activation after induction of hypertension and/or reduced beta mediated vasodilation. In a subsequent study, Spranger et al. (2017) concluded that during metaboreflex activation in hypertensive canines there is enhanced vasoconstriction of the coronary vasculature which limits increases in ventricular function. When this restraint of coronary blood flow was blocked via prazosin, the increases in coronary blood flow and CO were restored toward normal. These data indicate that the primary reason for the attenuated metaboreflex responses seen in the canine studies was enhanced coronary vasoconstriction which limited increases in CO. Inasmuch as the rise in CO is the primary mechanism mediating the metaboreflex response when activated during exercise, when the cardiac component was decreased, the pressor response was likewise attenuated (Sala-Mercado et al., 2013; Spranger et al., 2015, 2017). Additionally, in HF previous studies have shown that muscle metaboreflex activation induces limitations in coronary blood flow resulting in a further reduced capacity to increase CO to match physiological demands (Kaur et al., 2015a). Inasmuch as the responses in hypertension coincide with those observed in HF, the mechanism ultimately resulting in the profound pressor response in hypertension may have the same functional origin. Whether the metaboreflex is increased or attenuated may be dependent on which response is used for analysis, e.g., when a shift in mechanisms occurs (from CO based pressor response to peripheral vasoconstriction as occurs in HF), the strength of the metaboreflex in the control of CO is reduced but the strength of this reflex in the ability to elicit vasoconstriction is enhanced.

## Arterial Baroreflex

The arterial baroreceptor reflex is the primary reflex for beat-by-beat control of arterial pressure. In normotensive individuals during dynamic exercise many studies have shown that the operating point of the stimulus – response relationship for the arterial baroreflex is shifted to the right but the gain remains the same (Rowell and O’Leary, 1990; Papelier et al., 1994; O’Leary, 1996, 2006; Michelini et al., 2015). At rest, hypertensive individuals have reduced baroreflex control of HR (Judy and Farrell, 1979; Souza et al., 2008). Whether baroreflex control of sympathetic input to the peripheral vasculature is altered in hypertension is controversial (Sapru and Wang, 1976; Judy and Farrell, 1979; Thames et al., 1984; Matsukawa et al., 1991a,b; Dampney et al., 2005; Rossi et al., 2010). To our knowledge no study has systematically examined the effect of exercise on the strength and mechanisms of the arterial baroreflex during exercise in hypertension. Legramante et al. (1999) did observe the effect of changes in posture on the responses to handgrip in an older cohort of normotensive and hypertensive men and found that the effect of the static exercise on spontaneous baroreflex control of heart period was unaffected by the posture and was similar between normotensive and hypertensive groups. Inasmuch as posture changes unloads cardiopulmonary and arterial baroreceptors to variable extents, it is difficult to conclude whether the baroreflex is altered during exercise in hypertensive subjects. The authors attributed the lack of effect of hypertension on the baroreflex responses at rest to perhaps the age of the subjects. Some studies have investigated whether exercise training affects the baroreceptor reflex in hypertension but none have shown whether the arterial baroreflex strength and mechanisms are different between normotensive and hypertensive subjects during exercise. Burger et al. (1998) showed that in female exercise trained SHR, the baroreflex operating point of the systolic pressure – HR relationship was shifted to a higher point and the gain was reduced with increasing workloads, however, responses from normotensive animals were not investigated in that study. Moraes-Silva et al. (2010) demonstrated that treadmill training in during hypertension reduced absolute levels and the variability of blood pressure and HR when compared to sedentary hypertensive rats. Since a normotensive group was not utilized in the experiments previously mentioned, it is difficult to draw any conclusions about the arterial baroreflex function during exercise in hypertension. Furthermore the methods used to assess baroreceptor reflex activity is not consistent between studies, this provides a possible reason for the mismatch between the findings in the studies that have been done (O’Leary, 1996). Therefore further studies with appropriate controls are needed to fully understand impact of hypertension on arterial baroreflex control during exercise.

## Baroreflex – Metaboreflex Interaction in Hypertension

The arterial baroreflex resets during exercise to a higher set point in normotensive subjects (Rowell and O’Leary, 1990; Papelier et al., 1994; O’Leary, 1996, 2006; Michelini et al., 2015). Even though the arterial baroreflex is at a higher set point it still restrains the rise in arterial pressure caused by the muscle metaboreflex during dynamic exercise under normotensive conditions (Sheriff et al., 1990; Kim et al., 2005b). Sheriff et al. (1990) observed that sino-aortic baroreceptor denervation (SAD) increased the rise in arterial pressure in response to muscle metaboreflex activation (induced via reductions in hindlimb blood flow) during mild treadmill exercise in dogs. These results indicate that the arterial baroreflex buffers the muscle metaboreflex by about 50%. Subsequently, Kim et al. (2005b) showed that this buffering is due to arterial baroreflex attenuation of metaboreflex-induced peripheral vasoconstriction inasmuch as after SAD pronounced vasoconstriction now occurred along with the substantial increases in CO during metaboreflex activation. Therefore, the baroreflex nearly completely prevents the metaboreflex from causing substantial peripheral vasoconstriction. As workload rises, the skeletal muscle vasculature progressively becomes the largest fraction of the total vascular conductance and therefore substantial pressor responses via peripheral vasoconstriction could only occur via constriction of the active muscle (O’Leary, 1991; Kaur et al., 2016, 2018). This could engender a positive feedback, vicious cycle where further metaboreflex activation causes further skeletal muscle vasoconstriction. Indeed, recent studies have shown that there is some vasoconstriction within the active skeletal muscle during metaboreflex activation and this serves as an amplifier of the original response (Kaur et al., 2016). After induction of HF, this vasoconstriction in skeletal muscle is substantially greater perhaps due to depressed ability of the baroreflex to buffer metaboreflex-induced peripheral vasoconstriction (Kim et al., 2005a; Kaur et al., 2018). During exercise, hypertensive individuals experience accentuated increases in arterial blood pressure. Whether this is due to attenuated baroreflex restraint of pressor responses to activation of the muscle metaboreflex is unknown. Further studies are need to determine whether there is diminished arterial baroreflex function in hypertension which may be a potential mechanism allowing for the exaggerated increases in blood pressure during dynamic exercise.

## Conclusion

In summary, it is known that central command, skeletal muscle reflexes and the arterial baroreflex are important for regulating the cardiovascular system during exercise. To what extent hypertension alters these individual mechanisms and the interaction between these reflexes is not well-understood as some studies lack appropriate controls. Also since multiple models are utilized (i.e., humans, rats, and dogs), resting and locomotive postural position may be play a role in the sometimes disparate results. Therefore, more studies are needed to understand how hypertension alters neural control of cardiovascular function which allows for the often marked increases in arterial pressure observed in these patients during exercise (Gibbons et al., 2002). In particular how hypertension affects the central neural processes involved in integrative cardiovascular control during exercise in not understood and could be a target of therapeutic strategies.

## Author Contributions

MD wrote the first draft of the manuscript. DO’L and JM wrote sections of the manuscript. All authors contributed to manuscript revision, read and approved the submitted version.

## Conflict of Interest Statement

The authors declare that the research was conducted in the absence of any commercial or financial relationships that could be construed as a potential conflict of interest.
